# A single crystalline porphyrinic titanium metal–organic framework†
†Electronic supplementary information (ESI) available. CCDC [1036868]. For ESI and crystallographic data in CIF or other electronic format. See DOI: 10.1039/c5sc00916b



**DOI:** 10.1039/c5sc00916b

**Published:** 2015-04-28

**Authors:** Shuai Yuan, Tian-Fu Liu, Dawei Feng, Jian Tian, Kecheng Wang, Junsheng Qin, Qiang Zhang, Ying-Pin Chen, Mathieu Bosch, Lanfang Zou, Simon J. Teat, Scott J. Dalgarno, Hong-Cai Zhou

**Affiliations:** a Department of Chemistry , Texas A&M University , College Station , Texas 77842-3012 , USA . Email: zhou@chem.tamu.edu; b Advanced Light Source , Lawrence Berkeley National Laboratory Berkeley , CA 947240 , USA; c Institute of Chemical Sciences , Heriot-Watt University Riccarton , Edinburgh EH14 4AS , UK

## Abstract

We successfully assembled the photocatalytic titanium-oxo cluster and photosensitizing porphyrinic linker into a metal–organic framework (MOF), namely PCN-22. A preformed titanium-oxo carboxylate cluster is adopted as the starting material to judiciously control the MOF growth process to afford single crystals. This synthetic method is useful to obtain highly crystalline titanium MOFs, which has been a daunting challenge in this field. Moreover, PCN-22 demonstrated permanent porosity and photocatalytic activities toward alcohol oxidation.

## Abstract 2

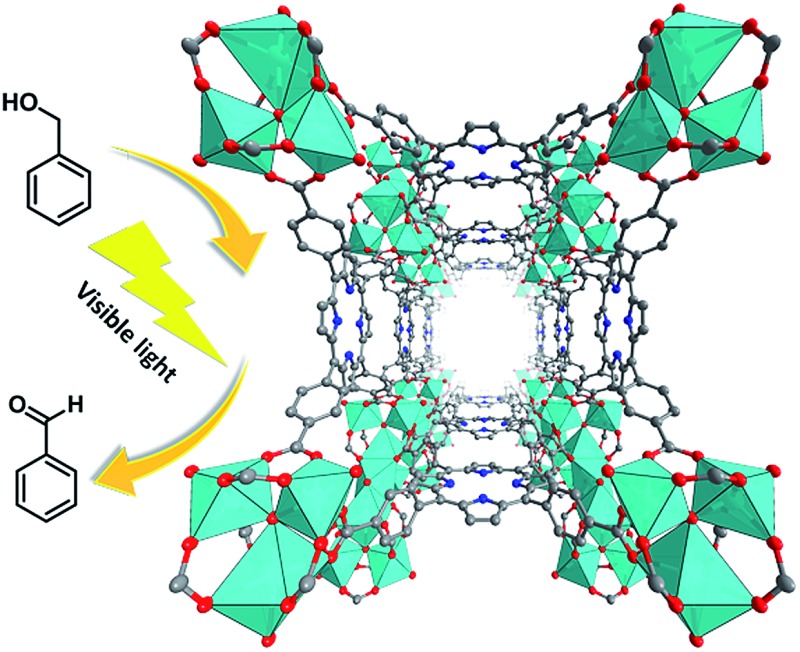
We have successfully synthesized a single crystalline porphyrinic titanium MOF, namely PCN-22. PCN-22 represents an important step towards mimicking dye sensitized TiO_2_ in MOFs.

## Introduction

Metal–organic frameworks (MOFs) are a promising class of highly ordered porous materials with potential applications in gas storage, catalysis, and photoelectric devices.^1–8^ For heterogeneous catalytic processes, the accessible external surface of the solid-state catalyst usually plays a decisive role on the catalytic efficiency.^9,10^ MOFs provide a platform to synthesize new heterogeneous catalysis with highly accessible external and internal surface and evenly distributed active sites.^11^ In addition, MOFs offer a bottom-up approach to tune their structures and functionalities by judicious design of inorganic building units and/or organic linkers.^12–16^ In previous studies, the functionalization of MOFs has mainly relied on the modification of organic linkers, whereas the functionalization of inorganic nodes has not been well explored thus far, except for utilization of open metal sites as Lewis acid for catalysis.^17–22^ The main reason for this is that catalytic processes involving labile coordinated metal centers can potentially compromise the structural integrity of the MOFs. Therefore, constructing a stable MOF with reactive metal nodes poses a great challenge. The development of titanium MOF MIL-125 represents an important breakthrough in the utilization of metal nodes as functional moieties.^23^ The titanium-oxo clusters in MIL-125 not only form strong bonds with benzenedicarboxylate (BDC) linkers to afford a highly stable framework, but also endow the material with photocatalytic activity. Nevertheless, the BDC linker in MIL-125 did not contribute to the catalytic process. Efforts were made to functionalize the BDC linker with amino groups acting as photosensitizers in MIL-125; however, the incorporation of efficient photosensitizers for visible-light-induced catalysis remains a challenge.^24–26^

Porphyrin-sensitized TiO_2_ has been widely investigated as a photocatalyst due to its high catalytic efficiency and chemical stability.^27–31^ This inspired us to mimic dye-sensitized TiO_2_ in a MOF matrix. The titanium-oxo clusters can act as photoactive sites with the porphyrinic ligands acting as photosensitizers to extend the optical response of MOF into the visible region through a dye sensitized mechanism.^32^ In this way, the organic and inorganic components of MOF can work cooperatively, making it an integrated dye-sensitized photoactive system. By incorporating the porphyrin-sensitized TiO_2_ system into a highly ordered porous material, the resulting porphyrinic titanium MOF has the following advantages: first, the titanium-oxo clusters can be periodically arranged and separated by organic linkers on the molecular level, leading to a much higher accessible surface compared to that of bulk TiO_2_. Second, the visible light photo-responsiveness of the porphyrinic titanium MOF would be much more sensitive than that of MIL-125 and its derivatives because of the high efficiency of the porphyrin antenna. Meanwhile, the Ti^4+^ ion has a high *Z*/*r* value, which forms a strong electrostatic interaction with the carboxylate ligand, resulting in an ultra-stable framework. The exceptional stability and visible light photocatalytic activity should make porphyrinic titanium MOF a suitable platform for visible-light driven photocatalysis.

Despite all these merits, only two titanium MOFs (MIL-125 and NTU-9) have been reported previously.^23,33^ The underdevelopment of titanium MOFs is due to the difficulty in achieving high crystallinity. To obtain single crystals, a reversible bond association/dissociation process is required to allow sufficient structure reorganization and defect reparation. The strong Ti–O bond, however, makes the bond dissociation extremely difficult during MOF growth. Our previous work has demonstrated that the reversibility of the MOF crystallization process can be promoted by increasing the temperature, using metal clusters as precursors and carboxylic acids as competing reagents.^34^ With the adoption of a preformed titanium-oxo carboxylate cluster as the metal source, TCPP (TCPP = tetrakis(4-carboxyphenyl)porphyrin) as the organic linker and benzoic acid as a modulating reagent, a single crystalline titanium MOF has been successfully obtained, designated as PCN-22 (PCN = porous coordination network). To the best of our knowledge, this is the first single crystalline titanium MOF based on carboxylate linkers.^35^ This work is a breakthrough in the synthesis of highly crystalline titanium MOF, which would be conducive to the development of titanium MOF chemistry. Moreover, the single crystalline product provides an opportunity to thoroughly characterize the structure and understand the relationship between structure and properties, which will be instructive for future design and synthesis of functional titanium MOFs. Moreover, PCN-22 demonstrates an example of mimicking the porphyrin-sensitized TiO_2_ in a porous MOF matrix. It shows photocatalytic activities toward alcohol oxidation with extensive optical response in the visible-light region, illustrating the synergetic effect of combining inorganic and organic components in a tailored framework.

## Results and discussion

PCN-22 was prepared by the reaction of Ti_6_O_6_(O^i^Pr)_6_(abz)_6_ (abz = 4-aminobenzoate),^36^ TCPP and benzoic acid under solvothermal conditions. The preformed Ti_6_O_6_(O^i^Pr)_6_(abz)_6_ cluster was used as a starting material instead of the titanium salt, which not only slows down the crystallization process but also effectively diminishes the hydrolysis of Ti^4+^ ions, avoiding the formation of TiO_2_. Moreover, the air stable titanium-oxo carboxylate clusters were also easier to handle compared to titanium salts. When reactions were conducted with Ti(O^i^Pr)_4_ or TiCl_4_ instead of titanium-oxo clusters under identical conditions, no crystalline products were obtained. At the same time, an excess amount of benzoic acid was added as a competing reagent, which could further slow down the forward reaction during the MOF growth process, favoring the formation of crystalline products.

**Fig. 1:**
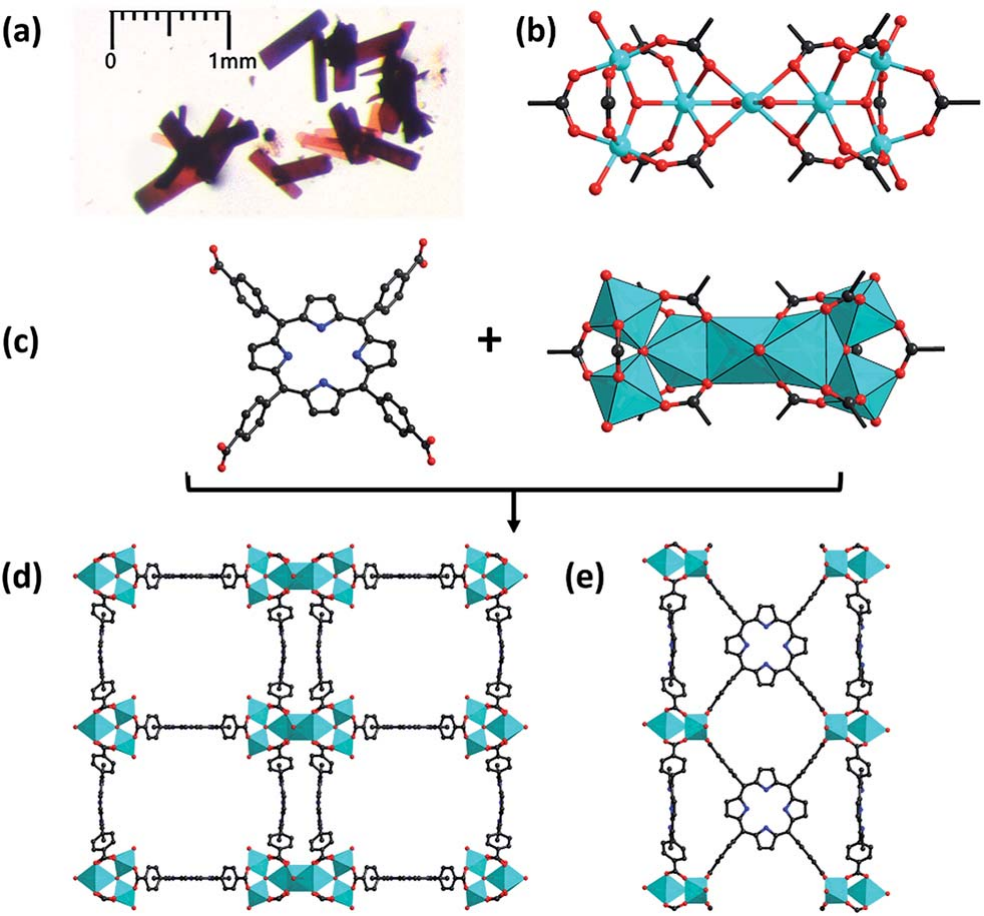
(a) Microscope image of PCN-22 crystals; (b) structure of the Ti_7_O_6_ cluster (only O atoms of DEF molecules are shown for clarity); (c) representation of the tetratopic TCPP linker (left) and 12-connected Ti_7_O_6_ cluster (right); (d) views of the structure of PCN-22 along *a*-axis and (e) *b*-axis. Color scheme: red O; black C; blue N; and cyan Ti. H atoms are omitted for clarity.

Single-crystal X-ray diffraction analyses revealed that PCN-22 crystalizes in the monoclinic *P*2/*m* space group. Three Ti atoms are jointed into a Ti_3_O_3_ cluster by a μ_3_-O^2–^ ion and six carboxylates. A pair of Ti_3_O_3_ clusters is further bridged by a Ti atom to form an unprecedented Ti_7_O_6_ cluster (Fig. 1b). Overall, the 12-connected Ti_7_O_6_ cluster is composed of seven Ti^4+^ ions, two μ_3_-O^2–^ ions, two terminal O^2–^ ions, two terminal OH^–^ ions and two bridging DEF molecules. Each Ti_3_O_3_ subunit is connected with six TCPP linkers to construct a two dimensional layer. The adjacent 2D layers are further linked by bridging Ti atoms, forming a 3D framework. A tetragonal channel with a diameter of ∼1.5 nm is observed in PCN-22. Topologically, each Ti_3_O_3_ can be regarded as a 6-connected node and a TCPP linker can be seen as a 4-connected node. The overall structure is simplified into a novel (4, 6) connected net with a point symbol of {4^4^·6^2^}_3_{4^9^·6^12^}_2_, which has never been reported to the best of our knowledge.^37,38^

**Fig. 2:**
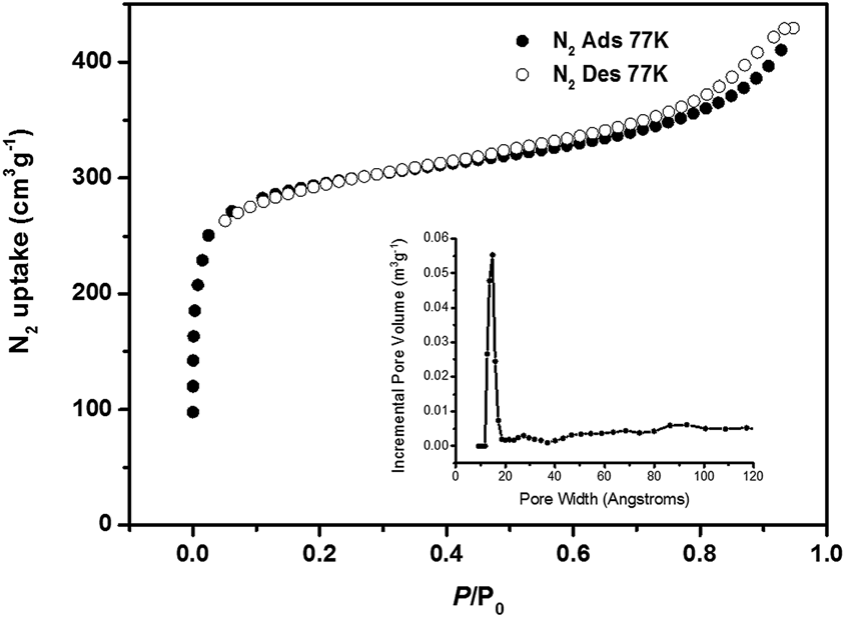
The N_2_ adsorption isotherm of PCN-22 at 77 K, 1 atm. The inset shows DFT pore size distribution of PCN-22 using data measured with N_2_ at 77 K.

The phase purity of PCN-22 was confirmed by comparison of the powder X-ray diffraction pattern and the simulated pattern from the crystal structure (Fig. S5†). The porosity of PCN-22 was examined by nitrogen sorption experiments at 77 K (Fig. 2). A N_2_ uptake of 430 cm^3^ g^–1^ (STP) and a Brunauer–Emmett–Teller (BET) surface area of 1284 m^2^ g^–1^ were observed for PCN-22. Density Functional Theory (DFT) calculations from the N_2_ sorption curve indicate that there is one type of pore with a diameter of 1.5 nm (Fig. S6†) assigned to the solvent accessible tetragonal channel, which is consistent with the crystallographic data when van der Waals radius is considered (Fig. S8†).

**Fig. 3:**
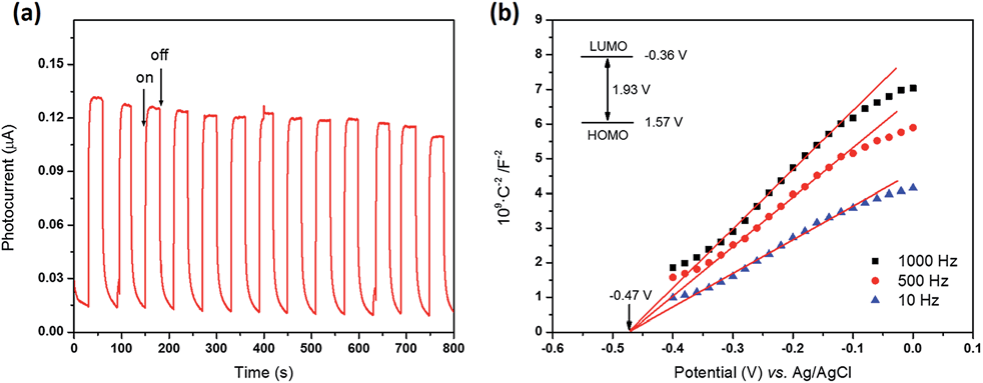
(a) Transient photocurrent responses of PCN-22 in 0.5 M Na_2_SO_4_ aqueous solution at a bias of 0.5 V *vs.* Ag/AgCl under visible light irradiation; (b) Mott–Schottky plot of PCN-22 in 0.5 M Na_2_SO_4_ aqueous solution.

PCN-22 was obtained as dark red crystals (Fig. 1a). The diffuse reflectance UV-vis spectrum of PCN-22 shows a broad range of absorption from 200 nm to 640 nm (Fig. S10†). The calculated band-gap of PCN-22 from the Tauc plot^39^ is 1.93 eV, which is smaller than the reported Ti-based MOFs MIL-125 (3.6 eV) and MIL-125-NH_2_ (2.6 eV).^24–26^ The photocurrent profile of PCN-22 indicates that this material is active under visible light (>450 nm) illumination (Fig. 3a). The Mott–Schottky measurement was performed to further reveal the flat-band potential of PCN-22. The positive slope of the obtained C^2–^ to potential plot is consistent with that of typical n-type semiconductors (Fig. 3b). It can be observed that the flat-band potential (*V*_fb_) of PCN-22 is –0.47 V *vs.* Ag/AgCl (–0.26 V *vs.* NHE). These results indicate that PCN-22 should be a suitable candidate for light harvesting and photo-induced catalysis.

**Scheme 1:**
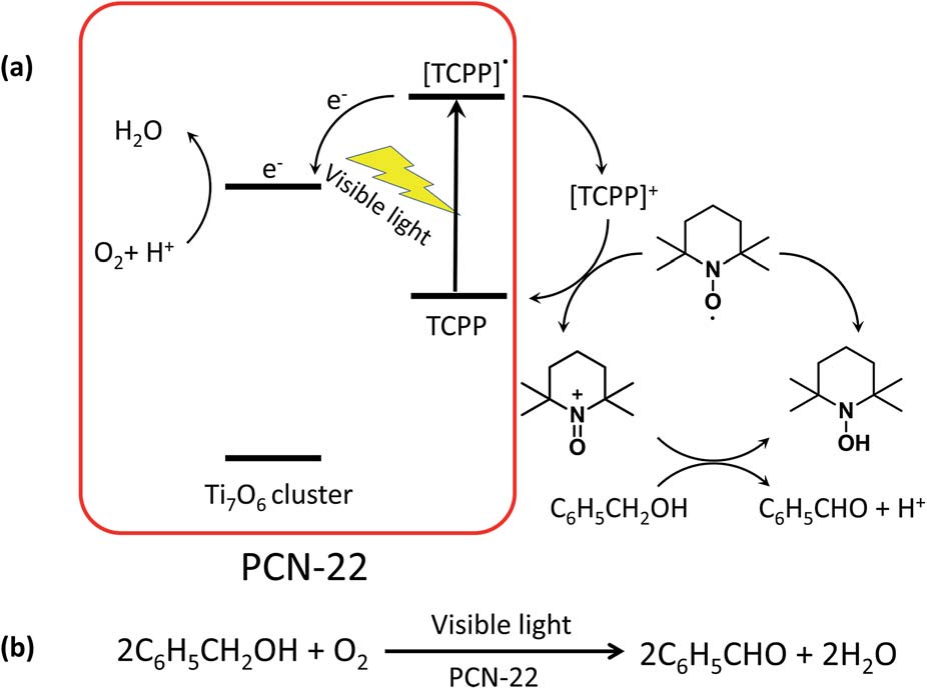
(a) Proposed mechanism for PCN-22/TEMPO system; (b) reaction catalyzed by PCN-22.

**Fig. 4:**
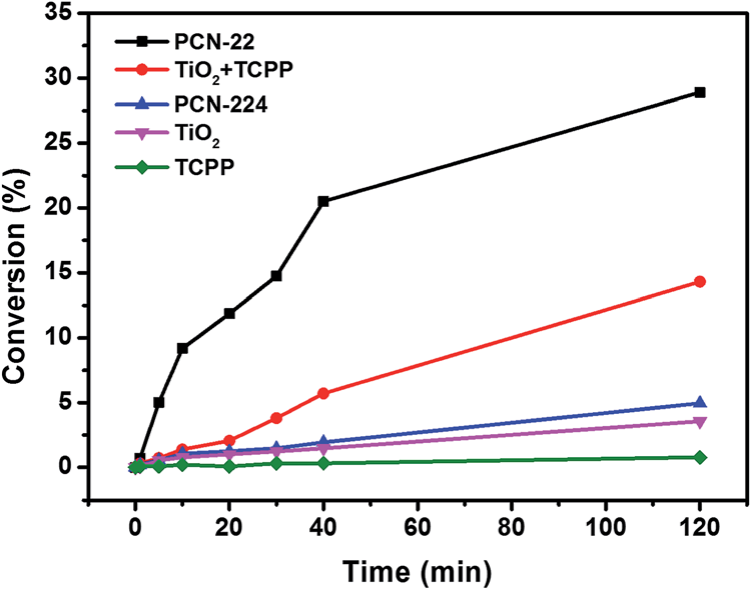
Time course of the benzyl alcohol oxidation reaction catalyzed by different catalyst.

In order to test the catalytic activity of PCN-22, we designed a PCN-22/TEMPO system (Scheme 1) for a photocatalyzed alcohol oxidation reaction (TEMPO = 2,2,6,6-tetramethylpiperidinyloxyl). According to the relevant research on a dye/TiO_2_/TEMPO system,^40^ we proposed a probable mechanism for the PCN-22/TEMPO system. The TCPP linkers are excited by visible light to inject electrons into Ti_7_O_6_ clusters, yielding [TCPP]^+^. Meanwhile, TEMPO is oxidized to TEMPO^+^ by [TCPP]^+^, which then selectively oxidizes alcohol into aldehyde by a two-electron-transfer mechanism. The conversion of a benzyl alcohol to the corresponding benzaldehyde reaches 28% under visible-light irradiation in two hours with high selectivity (almost 100%). The turnover number (TON) is over 100, indicating a catalytic process. Moreover, as a heterogeneous catalyst, PCN-22 can be easily recovered by centrifugation without obvious decrease in activity and selectivity after three successive runs (Table S2†). For comparison, the catalytic performances of TiO_2_, TCPP, a mechanical mixture of TiO_2_ and TCPP, as well as PCN-224 (ref. 41) (a previously reported zirconium porphyrinic MOF) were examined under the same conditions. As shown in Fig. 4, PCN-22 stands out to be the most efficient photocatalyst among these materials. PCN-224 is composed of Zr_6_O_8_ clusters and TCPP ligands. It contains a channel of 1.9 nm which is similar to that of PCN-22. However, PCN-224 shows low photocatalytic activity which can be attributed to the large energy gap and fast charge recombination of the Zr_6_O_8_ cluster. The low activity observed by the physical mixture of TiO_2_ and TCPP can be simply ascribed to the aggregation of the active sites. In contrast, the highly porous structure of PCN-22 makes each catalytic center accessible to the substrates. In addition, the titanium-oxo clusters in PCN-22 are periodically arranged and well separated by photosensitizers, which favors fast electron transfer from the photosensitizers to the titanium-oxo clusters and enhances the catalytic performance. The better performance of PCN-22 demonstrates that the incorporation of porphyrin sensitizer and titanium-oxo clusters into MOF matrix provides a new platform for the synthesis of photocatalysts.

## Conclusions

In summary, we report the first single crystalline titanium MOF, PCN-22, which was synthesized from preformed titanium-oxo carboxylate clusters and porphyrinic ligands. PCN-22 possesses high porosity and photocatalytic activity. It represents an important step towards mimicking dye sensitized TiO_2_ in MOFs, which will extend the potential applications of MOFs to clean energy generation.

## Experimental section

### Materials and instrumentation

Methyl-4-formylbenzoate was purchased from Oakwood Products, Inc. Pyrrole, propionic acid, titanium(iv)-isopropoxide (Ti(OCHCH_3_CH_3_)_4_), *N*,*N*′-diethylformamide (DEF), benzoic acid were purchased from Alfa Aesar. 5,10,15,20-Tetrakis(4-methoxycarbonylphenyl)porphyrin (TPPCOOMe) was prepared according to procedures described in S2.† All commercial chemicals were used without further purification unless otherwise mentioned.

Powder X-ray diffraction (PXRD) was carried out with a BRUKER D8-Focus Bragg–Brentano X-ray Powder Diffractometer equipped with a Cu sealed tube (*λ* = 1.54178 Å) at 40 kV and 40 mA. Thermogravimetric analyses (TGA) were carried out on a Shimadzu TGA-50 thermal analyzer from room temperature to 600 °C at a ramp rate of 5 °C min^–1^ in a flowing nitrogen atmosphere. Fourier transform infrared (IR) measurements were performed on a SHIMADZU IR Affinity-1 spectrometer. Nuclear magnetic resonance (NMR) data were collected on a Mercury 300 spectrometer. Samples were activated by supercritical carbon dioxide using MADRIDE prior to gas adsorption. Gas sorption measurements were conducted on a Micrometritics ASAP 2020 system. Energy dispersive X-ray spectroscopy was carried out by JEOL JSM-7500F with Oxford EDS system equipped with X-ray mapping.

### Synthesis

#### Ti_6_O_6_(O^i^Pr)_6_(abz)_6_

The Ti_6_O_6_(O^i^Pr)_6_(abz)_6_ cluster (Habz = 4-aminobenzoic acid) was synthesized according to a reported procedure.^34^ To a 2-propanol solution (6.0 mL) containing 4-aminobenzoic acid (192.1 mg, 1.40 mmol) added was titanium(iv) isopropoxide (103.6 μL). After stirring for 30 min at RT, the orange-colored slurry was heated to 100 °C for 77 h inside a sealed glass tube. The bright yellow crystalline product was collected, washed with 2-propanol and dried under vacuum for 3 h.

#### PCN-22

Ti_6_O_6_(O^i^Pr)_6_(abz)_6_ cluster (4 mg), TCPP ligand (10 mg), benzoic acid (100 mg) and DEF 2 mL were charged in a 4 mL Pyrex vial. The mixture was heated in 150 °C oven for 48 h. After cooling to room temperature, dark red, plate-like crystals were harvested.

### Photoelectrochemical studies

To prepare the working electrode, 10 mg PCN-22 was ground and dispersed in 2 mL acetone by sonication for 30 min. About 10 μL of the obtained slurry was coated on fluorine-doped tin oxide (FTO) glass with a fixed area of 0.5 cm^2^. The electrode was dried in air at 85 °C for 10 min. The photoelectrochemical tests were performed using an electrochemical workstation (CHI 830b). The photocurrent test was carried out using a three-electrode setup, in which the working electrode (PCN-22/FTO electrode) and the counter electrode (Pt plate electrode) were short-circuited. The 0.5 M Na_2_SO_4_ solution was used as the electrolyte. A 30 W fluorescent light bulbs was used as the visible light source. The Mott–Schottky curves were measured in dark using a three-electrode cell at frequencies of 10 Hz. The Pt plate was used as counter electrode and Ag/AgCl electrode (3 M KCl) was used as reference electrode. The flat band position (*V*_fb_) determined from the intersection is approximately –0.47 V *vs.* Ag/AgCl (*i.e.* –0.26 V *vs.* NHE) for PCN-22. Since it is generally believed that the bottom of the conduction band in many n-type semiconductors is more negative by about 0.10 V than the flat band potential, the conduction band (LUMO) of PCN-22 can be estimated to be –0.36 V *vs.* NHE. According to a band gap of 1.93 eV as described above, the valence band (HOMO) can be calculated to be 1.57 V *vs.* NHE.

### Alcohol oxidation reaction

The photocatalytic selective oxidation of benzoic alcohol was performed as follows. A mixture of benzyl alcohol (200 μL, 1.78 mmol), PCN-22 (10 mg) and TEMPO (3 mg, 0.02 mmol) in CH_3_CN was transferred into a 30 mL Pyrex bottle and purged with 30 mL min^–1^ O_2_ flow for 5 minutes. For controlled experiments, 10 mg of TiO_2_, TCPP, PCN-224, as well as a mechanical mixture of TiO_2_ (2 mg) and TCPP (8 mg) were used instead of PCN-22 under the same conditions. The suspension was continuously stirred and irradiated by a 300 W Xe lamp with a light filter to cut off light of wavelength <450 nm. Every 5 minutes, the reaction mixture was sampled with a 20 μL pipettor after 1, 5, 10, 20, 30, 40 and 120 minutes respectively. The samples were analyzed with HPLC (Shimadzu HPLC System with LC-20AD pump, SIL-20A Autoinjector and SPD-20A UV-vis Detector). The concentration of benzyl alcohol and benzaldehyde are calibrated by standard samples. The conversion and selectivity after two hours for three successive runs is shown in Table S2.† There was no obvious decrease in activity and selectivity after three successive runs, demonstrating a good recyclability of PCN-22 as a recyclable heterogeneous catalyst.
