# Supplementary material for: A single crystalline porphyrinic titanium metal–organic framework
Source: Chem Sci. 2015 Apr 28;6(7):3926–30. doi: 10.1039/c5sc00916b (PMC5707486; doi:10.1039/c5sc00916b)
Supplement: Supplementary file 1 [file SC-006-C5SC00916B-s001.pdf]

Electronic Supplementary Material (ESI) for Chemical Science.
This journal is © The Royal Society of Chemistry 2015

Supporting Information

A Single Crystalline Porphyrinic Titanium Metal−Organic Framework

Shuai Yuan[a]†, Tian-Fu Liu[a]†, Dawei Feng[a], Jian Tian[a], Kecheng Wang[a], Junsheng Qin[a], Qiang Zhang[a], Ying-Pin Chen[a], Mathieu Bosch[a], Lanfang Zou[a], Simon J. Teat,[b] Scott J. Dalgarno[c] and Hong-Cai Zhou [a]*

[a] Department of Chemistry, Texas A&M University, College Station, Texas 77842-3012, USA

[b] Advanced Light Source, Lawrence Berkeley National Laboratory Berkeley, CA 94720, USA

[c] Institute of Chemical Sciences, Heriot-Watt University Riccarton, Edinburgh EH14 4AS, U.K.

† Equal contribution to this work

*To whom correspondence should be addressed. Email: zhou@chem.tamu.edu
Tel: +1 (979) 845-4034; Fax: +1 (979) 845-1595

Contents

S1. Ligand Synthesis 3

S2. Syntheses of PCN-22 5

S3. X-ray Crystallography 6

S4. Topological Analyses 9

S5. N_2 Sorption Isotherm 10

S6. Simulation of the Accessible Surface Area 12

S7. Thermogravimetric Analysis 13

S8. Photoabsorption Performance 14

S9. Alcohol Oxidation Reaction 16

S10. SEM/EDX Analyses 18

S11. Acknowledgement 20

S1.Ligand Synthesis

The TCPP ligands (tetrakis(4-carboxyphenyl)porphyrin) were synthesized based on previous reports with slight modifications.[2] Typically, we obtained TCPP ligand by two steps as follows:

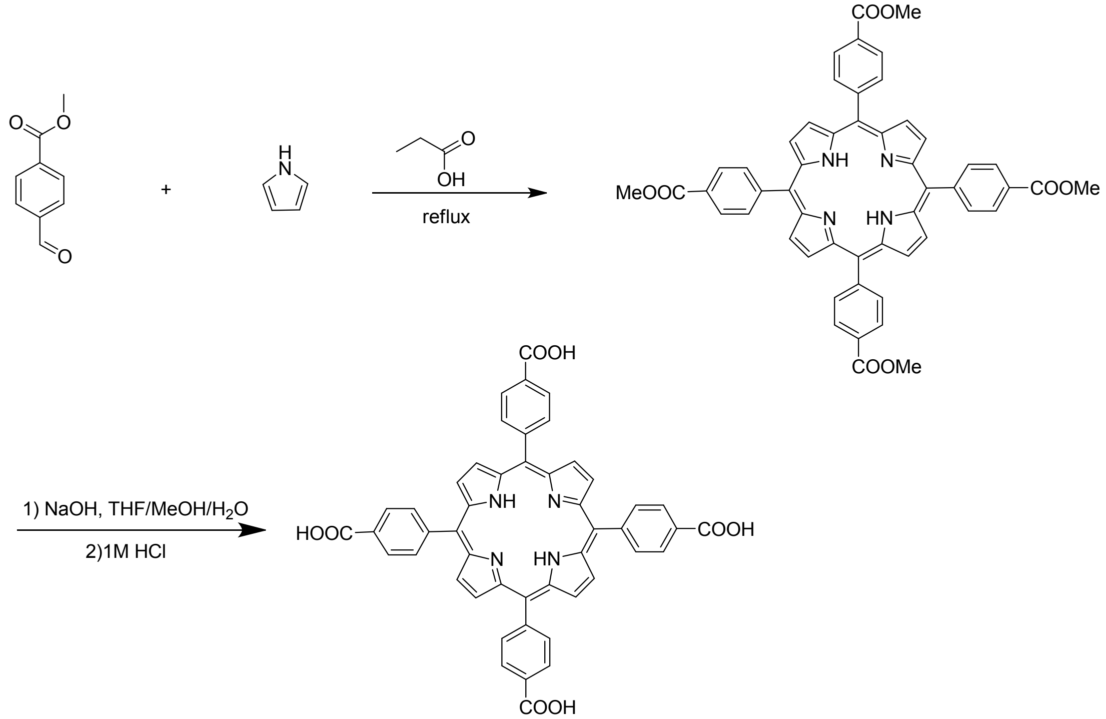

Scheme S1. Synthetic route for TCPP ligand.

(1) 5,10,15,20-Tetrakis(4-methoxycarbonylphenyl)porphyrin (TPPCOOMe)

In a 500-mL three necked flask, methyl *p*-formylbenzoate (6.9 g, 0.042 mol) was dissolved in propionic acid (100 mL). Pyrrole was then added dropwise (3.0 mL, 0.043 mol) and the solution was refluxed for 12 h. After the reaction, the mixture was cooled to room temperature, and the precipitates were collected by suction-filtration and washed with methanol, ethyl acetate and THF, respectively. After being dried in an oven for 12 hours, 1.9 g (2.24 mmol, 21% yield). Purple solid was obtained as pure product. 1H NMR (300 MHz, $CDCl_3$) δ 8.81 (s, 8H), 8.43 (d, 8H), 8.28 (d, 8H), 4.11 (s, 12H), 2.83 (s, 2H).

(2) Tetrakis(4-carboxyphenyl)porphyrin (TCPP)

The obtained ester (0.75 g) was stirred in 50 mL mixture of THF and MeOH (v : v = 1 : 1), to which a solution of NaOH (2.40 g, 60.00 mmol) in H_2O (25 mL) was introduced. This

mixture was refluxed for 12 h. After cooling to room temperature, THF and MeOH were evaporated. Additional water was added to the resulting water phase and the mixture was heated until the solid was fully dissolved. Then the homogeneous solution was acidified with 1M HCl until no further precipitate was detected. The precipitate was collected by filtration, washed with water and dried in vacuum oven. FTIR (KBr, cm^{-1}): ν= 3444 (m), 3034 (w), 2634 (w), 1702 (s), 1614 (s), 1570 (m), 1404 (s), 1311 (m), 1277 (s), 1204 (m), 1180 (m), 1106 (m), 1004 (s), 862 (m), 799 (s), 770 (s), 721 (m).

S2. Syntheses of PCN-22

$Ti_6O_6(O^iPr)_6(abz)_6$ cluster (4 mg), TCPP ligand (10 mg), benzoic acid (100 mg) and DEF 2mL were charged in a 4 mL Pyrex vial. The mixture was heated in 150 ºC oven for 48 h. After cooling to room temperature, dark red, plate-like crystals were harvested (1.5 mg, Yield. 20%). FTIR (KBr, cm^{-1}): 3637 (w), 1691 (w), 1606 (s), 1516(s), 1415 (vs), 1344 (m), 1178 (m), 1097 (w), 1018 (m), 966 (m), 800 (vs), 773 (m), 725 (m).

S3. X-ray Crystallography

The structure of PCN-22 was determined by single-crystal X-ray diffraction. The single crystal intensity data were collected at 110K on a D8 goniostat equipped with a Bruker APEXII CCD detector at the Advanced Light Source (Lawrence Berkeley National Laboratory) using synchrotron radiation (λ=0.67100 Å). The data frames were recorded using the program APEXII and processed using the *SAINT* routine within APEXII. The data were corrected for absorption and beam corrections based on the multi-scan technique as implemented in *SADABS*. The structures were solved by direct method using *SHELXS* and refined by full-matrix least-squares on F^2 using *SHELXL*[3] software. We obtained an R value of 0.1829 before *SQUEEZE* routine and 0.0953 after *SQUEEZE* routine of *PLATON*. The squeezed void volume was 4537 $Å^3$, equivalent to 61.6% of the unit cell. The contents of the solvent region are not represented in the unit cell contents in the crystal data. The asymmetric unit of PCN-22 was defined as seven Ti^{4+} ions, three deprotonated TCPP ligands, two μ_3-O, two O and two OH ions (O:OH=1:1), and two DEF solvent molecules. The TCPP was regarded as deprotonated molecules for charge neutrality. Also, the four terminal oxygens must be considered as half O^{2-} and half OH^{1-} ions for charge balance. Crystallographic data and structural refinements for PCN-22 were summarized in **Table S1**. Crystallographic data for the PCN-22 has been deposited with the Cambridge Crystallographic Data Centre (CCDC) under reference number CCDC 1036868. The data can be obtained free of charge via www.ccdc.cam.ac.uk/conts/retrieving.html.

Table S1. Crystal data and structure refinements for PCN-22.

	PCN-22
Formula	$Ti_7 C_{154} H_{96} N_{14} O_{32}$
	$[Ti_7(\mu_3\text{-}O)_2O_2(OH)_2] (TCPP)_3 (DEF)_2$
Formula weight	2989.75
Crystal Color	Dark red
Crystal System	Monoclinic
Space Group	*P*2/*m*
a (Å)	17.6073(6)
b (Å)	17.0188(7)
c (Å)	25.0871(10)
α (º)	90.000
β (º)	101.722(2)
γ (º)	90.000
V (Å^3)	7360.7(5)
Z	1
T (*K*)	110(2)
Wavelength (Å)	0.67100
$d_{calcd.}$ (g/cm^3)	0.674
μ(mm^{-1})	0.220
F(000)	1528
θ_{max} [deg]	23.29
Completeness	99.6%
Collected reflections	64509
Unique reflections	10998
Parameters	511
Restraints	3
R_{int}	0.0701
*R*1 [I > 2σ(I)]	0.0953
*wR*2 [I > 2σ(I)]	0.2222
*R*1 (all data)	0.1262
*wR*2 (all data)	0.2321
GOF on F^2	1.006
$\Delta\rho_{max}$/ $\Delta\rho_{min}$ [e·Å^{-3}]	0.978/-1.041

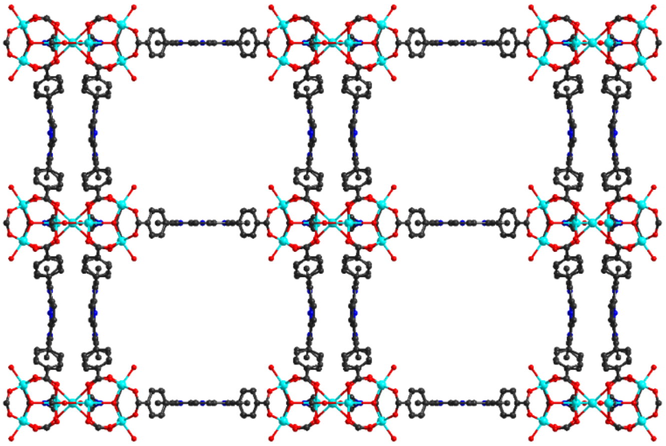

Figure S1. The crystal structure of PCN-22, viewed along *a* axis.

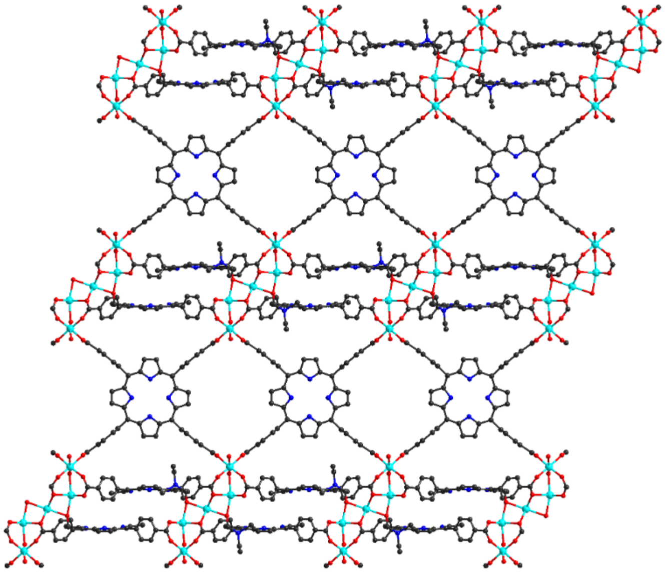

Figure S2. The crystal structure of PCN-22, viewed along *b* axis.

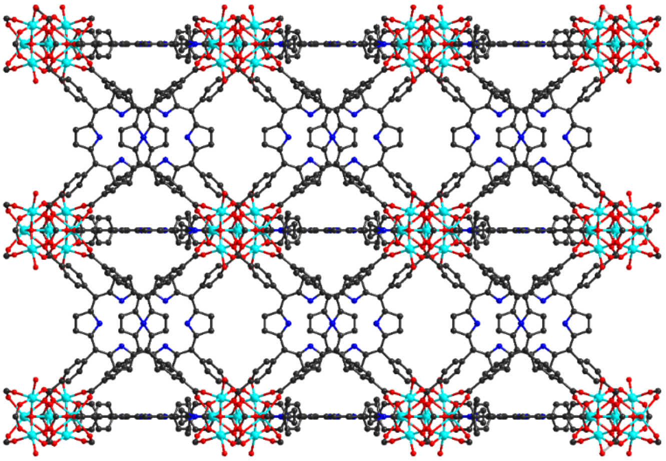

Figure S3. The crystal structure of PCN-22, viewed along *c* axis.

S4. Topological Analyses

The topological analyses of PCN-22 was performed by *TOPOS 4.0* Professional. The Ti_7O_6 clusters are composed of two Ti_3O_3 subunits bridged by one Ti^{4+} ion. Thus, each Ti_3O_3 can be regarded as a six connected node. The TCPP ligand can be viewed as a four connected node. The overall structure is simplified into a novel (4, 6) connected net with a point symbol of $\{4^4.6^2\}_3\{4^9.6^{12}\}_2$ (Figure S4).

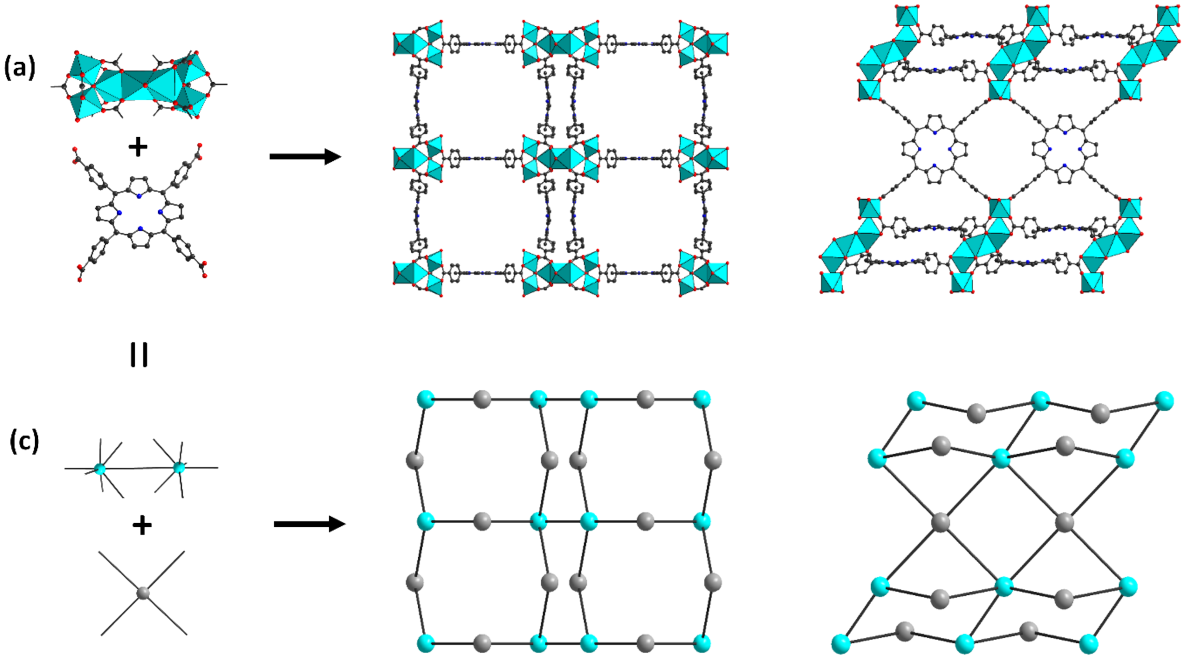

Figure S4. (a) Ball-and-stick representations of the Ti_7O_6 cluster, TCPP ligand and structure of PCN-22; (b) schematic representation of the (4, 6)-connected 3D network of PCN-22 in which Ti_3O_3 cluster and TCPP ligand are simplified as blue and gray nodes, respectively.

S5. N_2 Sorption Isotherm

Before the gas sorption experiment, as-synthesized PCN-22 samples were washed with $TiCl_4$/DEF solution (0.1 M) and then fresh DEF. Directly activated by evacuation, the samples failed to show permanent porosity due to the high surface tension caused by solvent evaporation. In order to eliminate the surface tension and gain access to the internal surfaces of the framework, we used the supercritical drying method to activate the sample where the solvent was first exchanged with liquid CO_2 and then heated above the carbon dioxide critical point (T = 31 °C, P = 73 atm). After holding for 30 min, the system was slowly vented to 1 atm. In this method, the elimination of surface tension prevents the pore collapse in the framework that would otherwise occur upon removal of solvent directly. Gas sorption measurements were then conducted using a Micrometritics ASAP 2020 system.

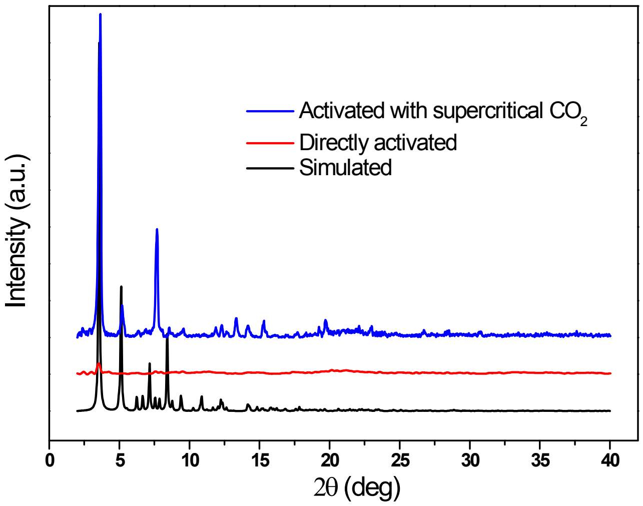

Figure S5. The PXRD of PCN-22 after direct activation (red), supercritical CO_2 activation (blue) and simulated pattern.

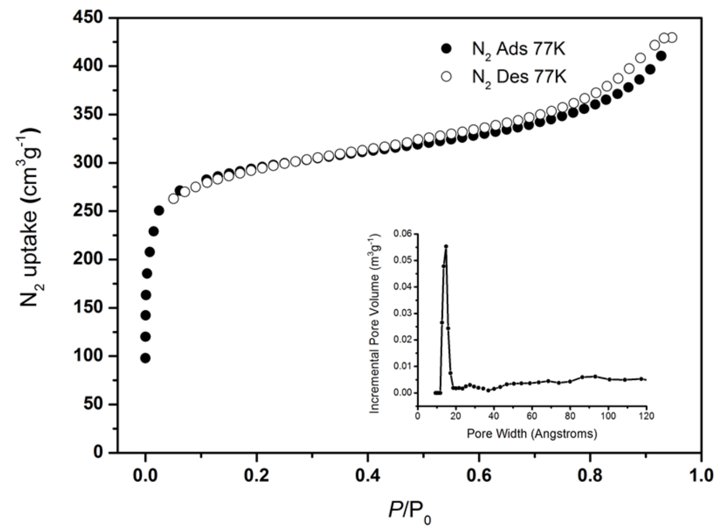

Figure S6. The N_2 adsorption isotherm of PCN-22 at 77 K, 1 atm. The inset shows DFT pore size distribution for PCN-22 derived by N_2 isotherm measured at 77 K, 1 atm.

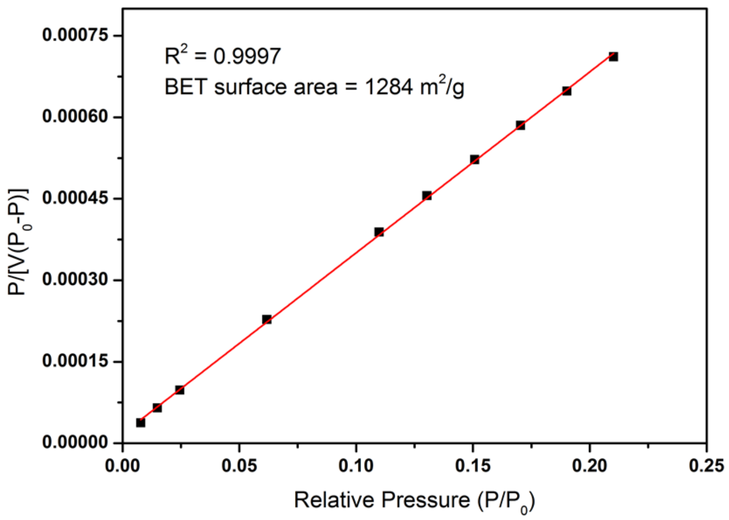

Figure S7. Plot of the linear region on the N_2 isotherm of PCN-22 using the BET equation.

S6. Simulation of the Accessible Surface Area

The accessible surface area of the PCN-22 solid was estimated using the strategy previously reported by Düren *et al.*[4] The surface area were calculated by *Materials Studio 6.0* software using a nitrogen sized probe molecules with a diameter of 3.68 Å and a grid interval of 0.25 Å.[5] The diameters of PCN-22 framework atoms were taken from the UFF force field: Ti= 2.83 Å, C= 3.43 Å, O= 3.12 Å, N= 3.26 Å, H= 2.57 Å.[6] The calculated surface area is 2045 m^3 g^{-1}, which is higher than the experimental surface area from N_2 sorption isotherm, indicating that the activation procedures could be further optimized. The accessible surface area is shown in **Figure S8**. It is clear that only the large tetragonal channel is accessible to N_2 molecules.

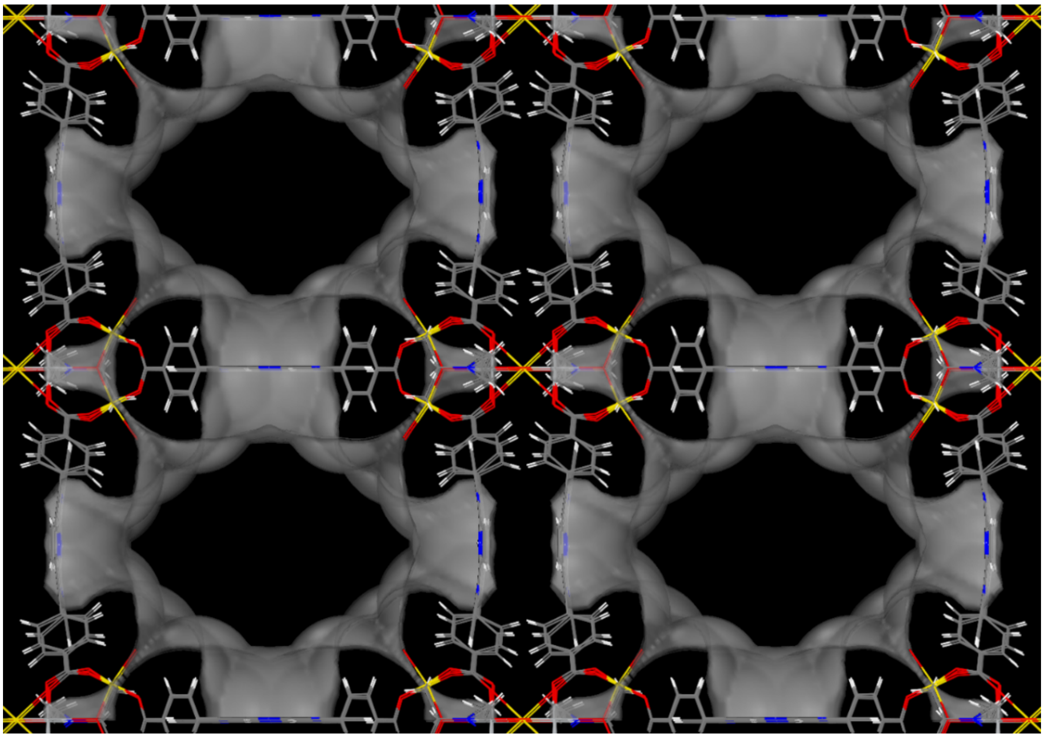

Figure S8: View of the accessible surface area (in grey) in the PCN-22 (color representation: red, O; black, C; Cyan, N, Yellow, White, H).

S7. Thermogravimetric Analysis

About 10 mg of PCN-22 sample was heated on a TGA-50 (Shimadzu) thermogravimetric analyzer from room temperature to 600 °C at a rate of 5 °C·min^{-1} under N_2 flow of 25 mL·min^{-1}. The decomposition temperature is around 350 °C.

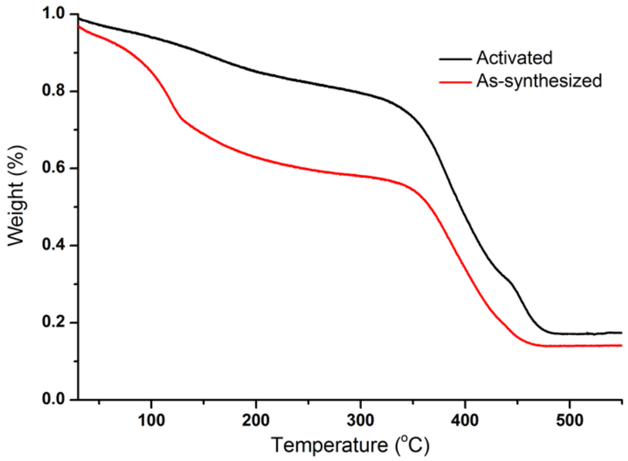

Figure S9. Thermogravimetric analysis of fresh and activated PCN-22.

S8. Photoabsorption Performance

The UV-vis spectrum was recorded on a Hitachi U-4100 UV-Vis-NIR spectrophotometer. As shown in Figure S10, the spectrum of PCN-22 shows a broad range of absorption in the visible region from 200 nm to 600 nm. The calculated optical band gap of PCN-22 derived from the absorption onset is 1.93 eV. It should be noted that PCN-22 is composed of discrete metal-oxo clusters which are separated by organic ligands. The interaction between each clusters is not strong enough to form a continuous band. This makes MOFs different from classical semiconductors. So the HOMO-LUMO gap terminology should be used in order to describe the discrete characteristics of the light-induced transitions in these coordination compounds.

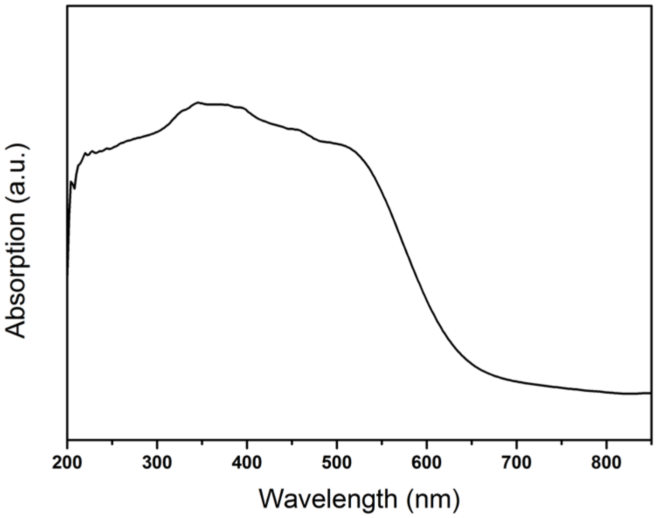

Figure S10. UV-vis spectrum of PCN-22.

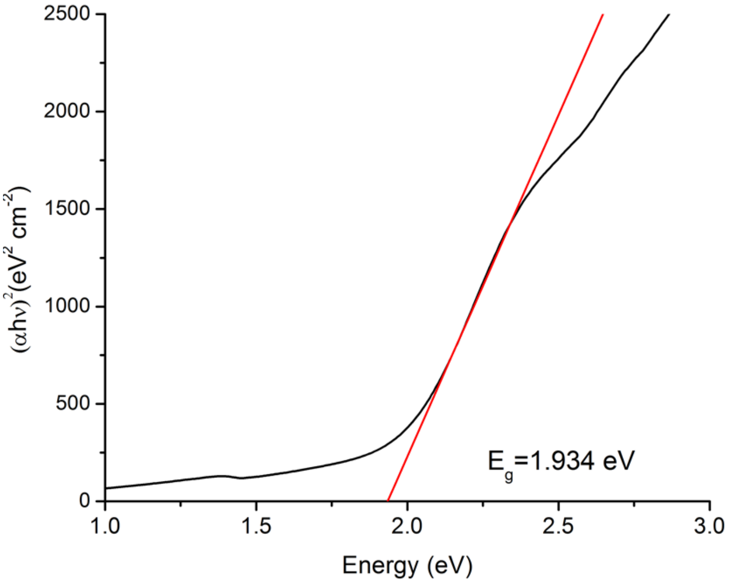

Figure S11. Band gap calculated from the UV-vis spectrum of PCN-22.

S9. Alcohol Oxidation Reaction

The conversion and selectivity after two hours for three successive runs is shown in Table S2. The PXRD pattern before and after catalytic reaction are shown in Figure S12. There was no obvious decrease in activity or selectivity after three successive runs and the PXRD pattern remains unaltered, demonstrating a good recyclability of PCN-22 as a recyclable heterogeneous catalyst.

Table S2. Catalytic recyclability of PCN-22/TEMPO for benzyl alcohol oxidation reaction for each recycling run

Reaction run	Conversion/%	Selectivity/%
1	28	>99
2	25	>99
3	25	>99

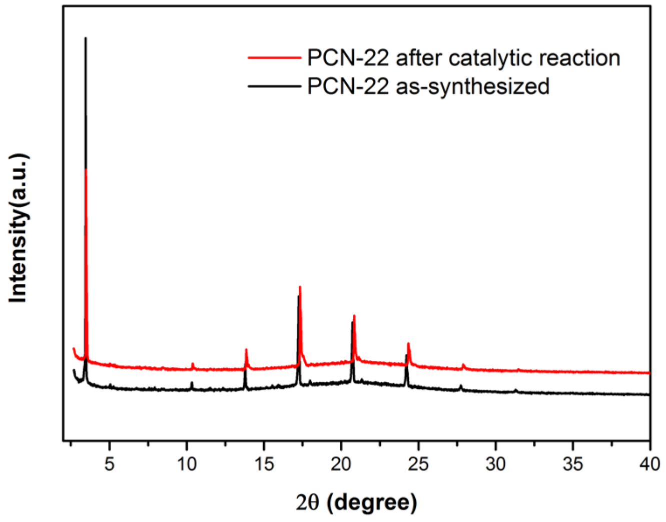

Figure S12. PXRD pattern for PCN-22 before and after catalytic reaction

S10. SEM/EDX Analyses

Instrumental information of scanning electron microscope (SEM) and energy dispersive X-ray spectroscopy (EDX) Analyses: Images and analyses of SEM/EDX were taken by FEI Quanta 600 FE-SEM. The Quanta 600 FEG is a field emission scanning electron microscope capable of generating and collecting high-resolution and low-vacuum images. It is equipped with a motorized x-y-z-tilt-rotate stage, providing the following movements: x = y = 150 mm (motorized); z = 65 mm (motorized); Tilt +70 degrees to –5 degrees (motorized); Source: Field emission gun assembly with Schottky emitter source. Voltage: 200 V to 30 kV Beam Current: >100 nA

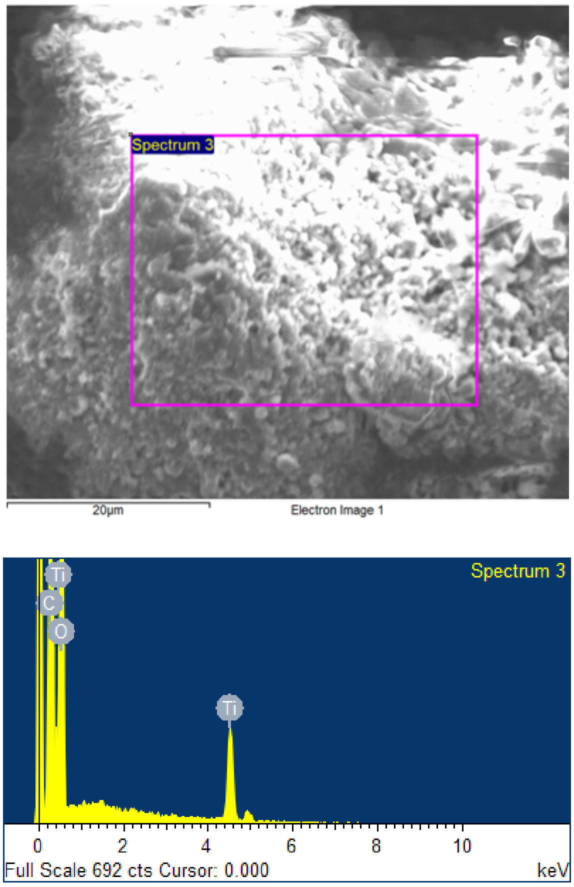

Figure S13. SEM/EDX analyses for PCN-22 after catalysis (sample prepared without coating).

Table S3. Atomic ratio in PCN-22.

Element	Weight%	Atomic%
C K	43.90	57.10
O K	37.82	36.93
Ti K	18.28	5.96
Totals	100.00	100

S11. Acknowledgement

S. Yuan and T.-F. Liu were supported by U. S. Department of Energy, Office of Science, Office of Basic Energy Sciences (DE-SC0001015), K. Wang and M. Bosch were supported by the U.S. Department of Energy, ARPA-e (DEAR0000249), Y.-P. Chen and D. Feng were supported by the Welch Foundation (A-1725), Q. Zhang was supported by the Office of Naval Research (N00014-14-1-0720). H.-C. Zhou gratefully acknowledges the support from U.S. Department of Energy, the Office of Naval Research, and the Welch Foundation.

References

[1] K. Hong, H. Chun, *Inorg. Chem.* **2013**, *52*, 9705-9707.

[2] N. Asano, S. Uemura, T. Kinugawa, H. Akasaka, T. Mizutani, *J. Org. Chem.* **2007**, *72*, 5320-5326.

[3] G. M. Sheldrick, *SHELXTL*, Version 6.14, Structure Determination Software Suite, Bruker AXS, Madison, WI, **2003**.

[4] T. Düren, F. Millange, G. Férey, K.S. Walton, R.Q. Snurr, *J. Phys. Chem. C*, **2007**, 111, 15350-15356.

[5] (a) C. Lastoskie, K.E. Gubbins, N. Quirke, *J. Phys. Chem.*, **1993**, 97, 4786-4796; (b) *Accelerys Materials Studio Release Notes*, Release 5.5.1; Accelrys Software, Inc.: San Diego, **2010**.

[6] A.K. Rappe, C.J. Casewit, K.S. Colwell, W.A. Goddard-III, W.M. Skiff, *J. Am. Chem. Soc.*, **1992**, 114, 10024-10035.
